# Roux-en-Y Gastric Bypass Surgery Induces Early Plasma Metabolomic and Lipidomic Alterations in Humans Associated with Diabetes Remission

**DOI:** 10.1371/journal.pone.0126401

**Published:** 2015-05-06

**Authors:** Tulika Arora, Vidya Velagapudi, Dimitri J. Pournaras, Richard Welbourn, Carel W. le Roux, Matej Orešič, Fredrik Bäckhed

**Affiliations:** 1 Wallenberg Laboratory and Sahlgrenska Center for Cardiovascular and Metabolic Research, Department of Molecular and Clinical Medicine, Institute of Medicine, University of Gothenburg, Gothenburg, Sweden; 2 VTT Technical Research Centre of Finland, Espoo, Finland; 3 Metabolomics Unit, Institute for Molecular Medicine Finland FIMM, Helsinki, Finland; 4 Department of Bariatric Surgery, Musgrove Park Hospital, Taunton, United Kingdom; 5 Diabetes Complications Research Centre, Conway Institute, School of Medicine and Medical Science, University College Dublin, Dublin, Ireland; 6 Gastrosurgical Laboratory, Sahlgrenska Academy, University of Gothenburg, Gothenburg, Sweden; 7 Steno Diabetes Center A/S, Gentofte, Denmark; 8 Novo Nordisk Foundation Center for Basic Metabolic Research, Section for Metabolic Receptology and Enteroendocrinology, Faculty of Health Sciences, University of Copenhagen, Copenhagen, Denmark; INRA, FRANCE

## Abstract

Roux-en-Y gastric bypass (RYGB) is an effective method to attain sustained weight loss and diabetes remission. We aimed to elucidate early changes in the plasma metabolome and lipidome after RYGB. Plasma samples from 16 insulin-resistant morbidly obese subjects, of whom 14 had diabetes, were subjected to global metabolomics and lipidomics analysis at pre-surgery and 4 and 42 days after RYGB. Metabolites and lipid species were compared between time points and between subjects who were in remission and not in remission from diabetes 2 years after surgery. We found that the variables that were most discriminatory between time points were decanoic acid and octanoic acid, which were elevated 42 days after surgery, and sphingomyelins (18:1/21:0 and 18:1/23:3), which were at their lowest level 42 days after surgery. Insulin levels were lower at 4 and 42 days after surgery compared with pre-surgery levels. At 4 days after surgery, insulin levels correlated positively with metabolites of branched chain and aromatic amino acid metabolism and negatively with triglycerides with long-chain fatty acids. Of the 14 subjects with diabetes prior to surgery, 7 were in remission 2 years after surgery. The subjects in remission displayed higher pre-surgery levels of tricarboxylic acid cycle intermediates and triglycerides with long-chain fatty acids compared with subjects not in remission. Thus, metabolic alterations are induced soon after surgery and subjects with diabetes remission differ in the metabolic profiles at pre- and early post-surgery time points compared to patients not in remission.

## Introduction

Roux-en-Y gastric bypass (RYGB) provides a successful treatment for long-term weight loss maintenance in morbidly obese patients [1]. RYGB is also associated with metabolic advantages such as improvement in glycemic control [2], which is observed before significant weight loss. Increased secretion of the incretin hormone glucagon-like peptide 1 and insulin following a test meal has been reported 1 week after gastric bypass [3,4]. However, although acute caloric restriction has been suggested to play a role in improved insulin resistance within a week of RYGB [5], it is not clear what other factors may influence these early changes. In addition, many patients, but not all, exhibit diabetes remission after surgery. The mechanisms and differences in the patients exhibiting diabetes remission are incompletely understood. Surgery is not without risk and predicting which patients may have the best results after surgery could help better personalize the evaluation of risks and benefits.

The significant gastrointestinal rearrangement associated with gastric bypass contributes to alterations in the metabolic and lipidomic status. Previous studies in rats have demonstrated reductions in urinary amines, cresols and tricarboxylic acid (TCA) intermediates after gastric bypass, suggesting effects on renal function and energy metabolism [6]. In humans, reductions in branched chain amino acids 1 month after RYGB have been shown to correlate with improvement in glucose homeostasis [7]. Furthermore, reductions in ceramides and nervonic acid have been reported 3–6 months after RYGB and have been shown to correlate negatively with improvement in insulin sensitivity after RYGB [8–10]. However, although metabolic improvements are observed as soon as 1 week after RYGB, it is not clear how early changes in the metabolic and lipidomic status are associated with these short-term improvements and with diabetes remission in the long term.

Here, we analyzed global metabolomic and lipidomic profiles of obese subjects with diabetes at pre-, 4 and 42 days after RYGB, and investigated which metabolites and lipid species correlated with insulin levels and could thus potentially contribute to metabolic improvements. We also compared metabolic profiles at pre-, 4 and 42 days after RYGB between subjects who were in remission (REM) with those who did not show diabetes remission (N-REM) 2 years after surgery to identify early differences in metabolites and lipid species that may contribute to the variation in diabetes remission.

## Materials and Methods

### Study cohort

The samples in the present study were obtained from a subgroup (16 subjects) of a previously studied cohort comprising 22 subjects [11]. All patients were insulin resistant as determined by their HOMA index; 14 were on treatment for diabetes, and 2 of these required insulin therapy. RYGB was performed on 16 subjects (5 men, 11 women) with a mean age of 47.4 ± 1.9 years and mean BMI of 48.9 ± 1.3 kg/m^2^. Patients had diabetes for at least 1 year and up to a maximum of 17 years. All patients underwent laparoscopic retrocolic antegastric RYGB (Roux limb 100 cm for BMI <50 kg/m^2^ and 150 cm for BMI ≥50 kg/m^2^). We used an enhanced recovery protocol and patients were allowed free fluids with a suggested intake of approximately 700 to 1000 kcal per day on return to the ward. By week 6, patients could have as varied a diet as tolerated. Patient characteristics are shown in Table 1 and further details are described in Pournaras *et al* 2010 [11].

**Table 1:** Clinical characteristic of all patients and patients in diabetes remission and not in remission.

	All patients^a^ (n = 16)	REM (n = 7)	N-REM (n = 7)
**Pre-gastric bypass**			
**Age (years)**	47.4±1.9	48.1±2.3	48.8±3.5
**Gender (male/female)**	5/11	4/3	0/7
**Weight (kg)**	140.4±4.5	152.5±4.6	130.4±4.2
**BMI (kg/m** ^**2**^ **)**	48.9±1.3	51.0±2.3	47.2±1.6
**Hb1Ac (%)**	8.5±0.5	9.1±0.7	7.9±0.8
**Fasting glucose (mmol/l)**	7.1±0.4	7.3±0.4	7.3±0.7
**Insulin dependent**	2/16	2/7	0/7
**Duration of diabetes (years)**	7.5±1.6 (1–17)	8.1±2.6 (1–17)	6.8±1.8 (5–16)
**24 months post-gastric bypass**			
**Weight loss (kg)**	43.1±3.4	39.9±3.0	46.5±6.7
**Weight loss (%)**	30.5±2.0	30.3±4.2	30.7±2.2
**BMI reduction (%)**	31.3±2.1	30.5±4.4	32.0±2.3
**Effective weight loss (%)**	62.6±3.9	59.4±7.1	66.1±5.5
**Hb1Ac (%)**	5.8±0.2	5.8±0.5	5.8±0.1
**Fasting glucose (mmol/l)**	5.7±0.5	4.9±0.2	6.4±0.9

Values are shown as mean±SEM (range). BMI = body mass index; HOMA = Homeostatic model assessment; HbA1c = Acylated hemoglobin 1c; REM = patients in diabetes remission; N-REM = patients not in diabetes remission

^a^All patients include 2 patients who did not have diabetes before surgery and were therefore not included in the REM or N-REM subgroups.

Fasting plasma samples were collected pre-surgery and 4 and 42 days after surgery. The changes at 4 days after surgery represent the combined effects of the surgical process and calorie restriction, whereas the profiles at 42 days after surgery may be indicative of a comparatively more stable physiological state when surgical stress has reduced and food intake is no longer restricted by externally imposed diets. All patients gave written and informed consent. Exclusion criteria included pregnancy, substance abuse, and more than 2 alcoholic drinks per day. The study was performed according to the principles of the Declaration of Helsinki and approved by the Somerset Research and Ethics committee (LREC Protocol Number: 05/Q2202/96).

Diabetes remission and weight loss were monitored for 2 years after RYGB. All patients completed the study and follow-up. Diabetes remission was defined, according to the American Diabetes Association guidelines, as a return to normal measures of glucose metabolism (HbA1c <6%, fasting glucose <5.6 mmol/l) at least 1 year after surgery without hypoglycemic pharmacologic therapy or ongoing procedures [2,11]. We used 2 years as the cut-off for early remission as described previously for short-term outcomes [12,13]. Of the 14 patients with diabetes before surgery, 7 were in diabetes remission 2 years after surgery.

### Metabolomic and lipidomic analysis

Metabolites were extracted from 15 μl of plasma samples that were combined with 10 μl of an internal standard mix (C17:0 94 mg/l; valine-d 19 mg/l; succinic acid-d4 32 mg/l) and 200 μl of methanol, vortexed for 2 min and incubated for 30 min at room temperature. The supernatant was separated and dried under constant flow of nitrogen and derivatized [14]. Metabolite analysis was carried out on a LECO Pegasus 4D instrument (Agilent Technologies) consisting of Agilent gas chromatograph combined with time-of-flight mass spectrometer (GCxGC-TOFMS).

For lipids, 10 μl of plasma samples were diluted with 10 μl of 0.9% NaCl and spiked with 20 μl of internal standard mix. The samples were subsequently extracted with 100 μl of chloroform-methanol (2:1) solvent, 60 μl aliquot from the separated lower phase was mixed with 10 μl of a labeled standard mixture [10 μg/ml glycerophosphatidylcholine (16:0/16:0-D3), glycerophosphatidylcholine (16:0/16:0-D6) and triglyceride (16:0/16:0/16:0-^13^C3)] and 0.5–1.0 μl was used for analysis. The major lipid classes (monoacylglycerols and phospholipids, diacylglycerols and glycerophospholipids, sphingolipids, triacylglycerols, and cholesteryl esters) were measured within a single sample run. A detailed technical description about the method has been described elsewhere [15]. Lipid extracts were analyzed on a Q-ToF Premier mass spectrometer (Waters) combined with an Acquity ultra performance liquid chromatography/MS (UPLC/MS).

### Statistical analysis

Data were analyzed using a web-based comprehensive metabolomics data processing tool, MetaboAnalyst 2.0 (http://www.metaboanalyst.ca) [16,17]. To determine the maximum separation among groups, a supervised multivariate regression technique, partial least squares discriminant analysis (PLS-DA), was performed on autoscaled data (i.e. mean-centered and divided by the standard deviation of each variable). Spearman rank correlation was performed for correlating metabolites and lipid species with insulin levels at 4 and 42 days after surgery. Unpaired two-tailed t-test was performed to compare metabolites and lipid species between REM and N-REM subjects using GraphPad Prism Software.

## Results

**Fig 1:**
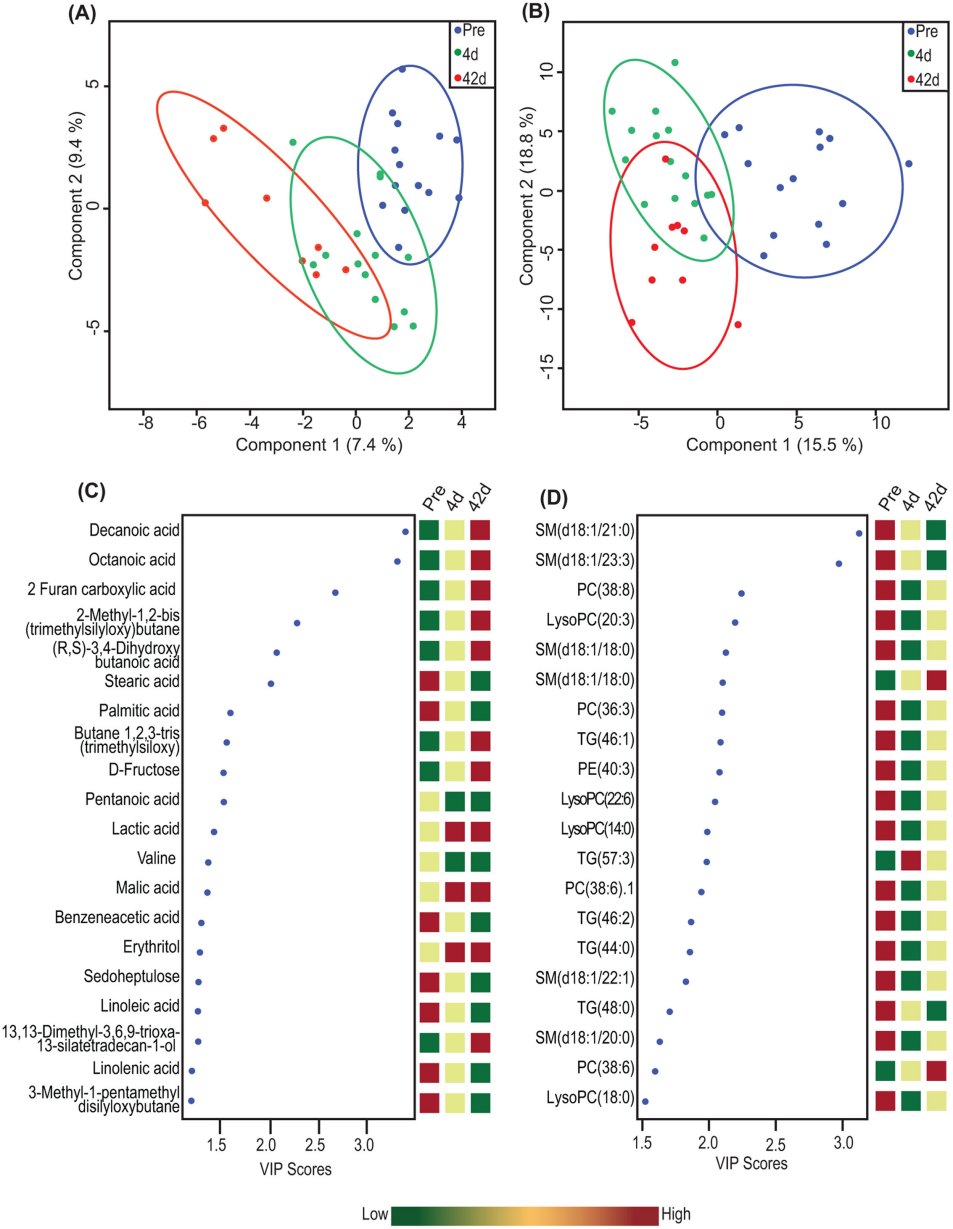
Partial least square discriminant analysis of lipidomic and metabolomic profiles of the subjects (n = 16). (A) Score plot showing clustering of subjects based on metabolomic and (B) lipidomic profiles at pre-surgery and 4 and 42 days after surgery. (C) Top 20 metabolites and (D) lipid species with their variable importance in projection (VIP) scores and relative abundance at pre-surgery and 4 and 42 days after surgery. LysoPC = lysophosphatidylcholine; PC = phosphatidylcholine; PE = phosphatidylethanolamine; SM = sphingomyelin; TG = triglycerides.

We identified 96 metabolites and 192 lipid molecular species in the plasma of patients before surgery and 4 and 42 days after surgery. PLS-DA showed that the metabolites and lipid species clustered according to time point rather than by subject, and samples obtained at 4 days after surgery were located between the samples taken pre-surgery and 42 days after surgery (Fig 1A and 1B). At 4 days after surgery, the relative levels of 15 out of 20 metabolites were intermediate of levels obtained pre-surgery and 42 days after surgery (Fig 1C), whereas, the relative levels of 14 out of 20 lipid species were at their lowest levels at 4 days after surgery (Fig 1D).

**Fig 2:**
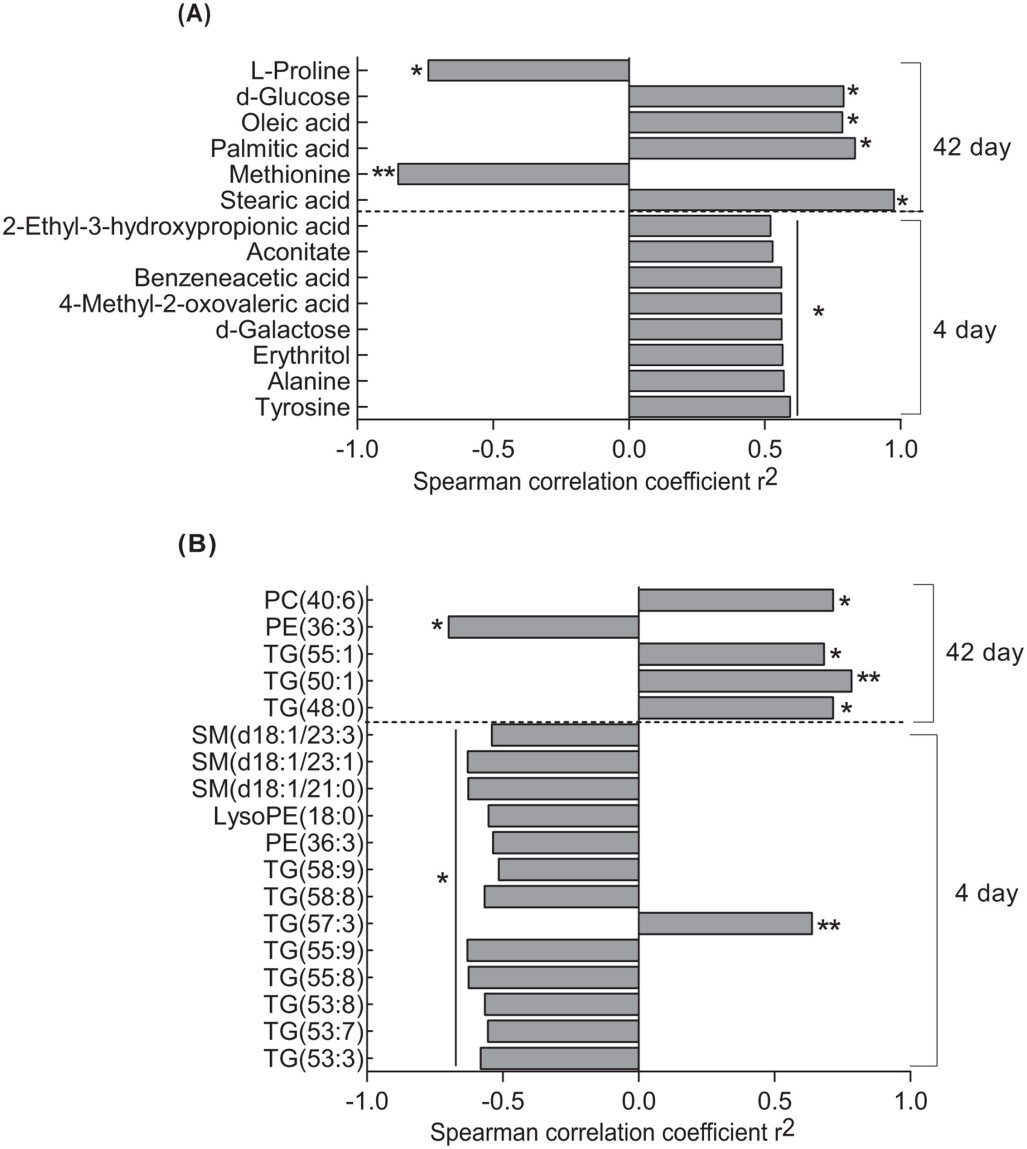
Spearman rank correlations of (A) metabolites (B) lipid species with insulin levels at 4 and 42 days after RYGB. *p<0.05; **p<0.01. LysoPE = lysophosphatidylethanolamine; PC = phosphatidylcholine; PE = phosphatidylethanolamine; SM = sphingomyelin; TG = triglycerides.

Insulin levels were lower at 4 and 42 days after surgery (10.3 ± 1.4 IU and 11.8 ± 2.3 IU, respectively) compared with pre-surgery levels (18.4 ± 3.1 IU). Insulin levels correlated positively with metabolites of BCAA and aromatic amino acids at 4 days after surgery and with stearic, palmitic and oleic acids at 42 days after surgery (Fig 2A). Among the lipids, SM species and TGs with long-chain fatty acids except TG (57:3) correlated negatively with insulin levels at 4 days, whereas other TG species (48:0, 50:1, 55:1) correlated positively with insulin levels at 42 days (Fig 2B). Metabolites such as stearic acid (r^2^ = 0.6, p = 0.02), TG (48:0; r^2^ = 0.6, p = 0.02) and TG (55:1; r^2^ = 0.6, p = 0.03) also correlated positively with insulin levels at pre-surgery.

Of the 14 patients with diabetes before surgery, 7 were in diabetes remission 2 years after surgery. Fasting glucose was lowered in all patients but showed a greater reduction in patients in remission than in those not in remission 2 years after surgery (Table 1). However, similar improvements in BMI, percentage weight loss and Hb1Ac levels were observed in patients in remission and not in remission 2 years after surgery (Table 1). The similar improvement in Hb1Ac levels in both groups of patients indicates that all patients had good glycemic control but patients in diabetes remission achieved this without medication (and can thus be defined as in remission). Of the 7 patients not in remission, 5 were on metformin, 1 was on gliclazide and 1 was on pioglitazone.

**Fig 3:**
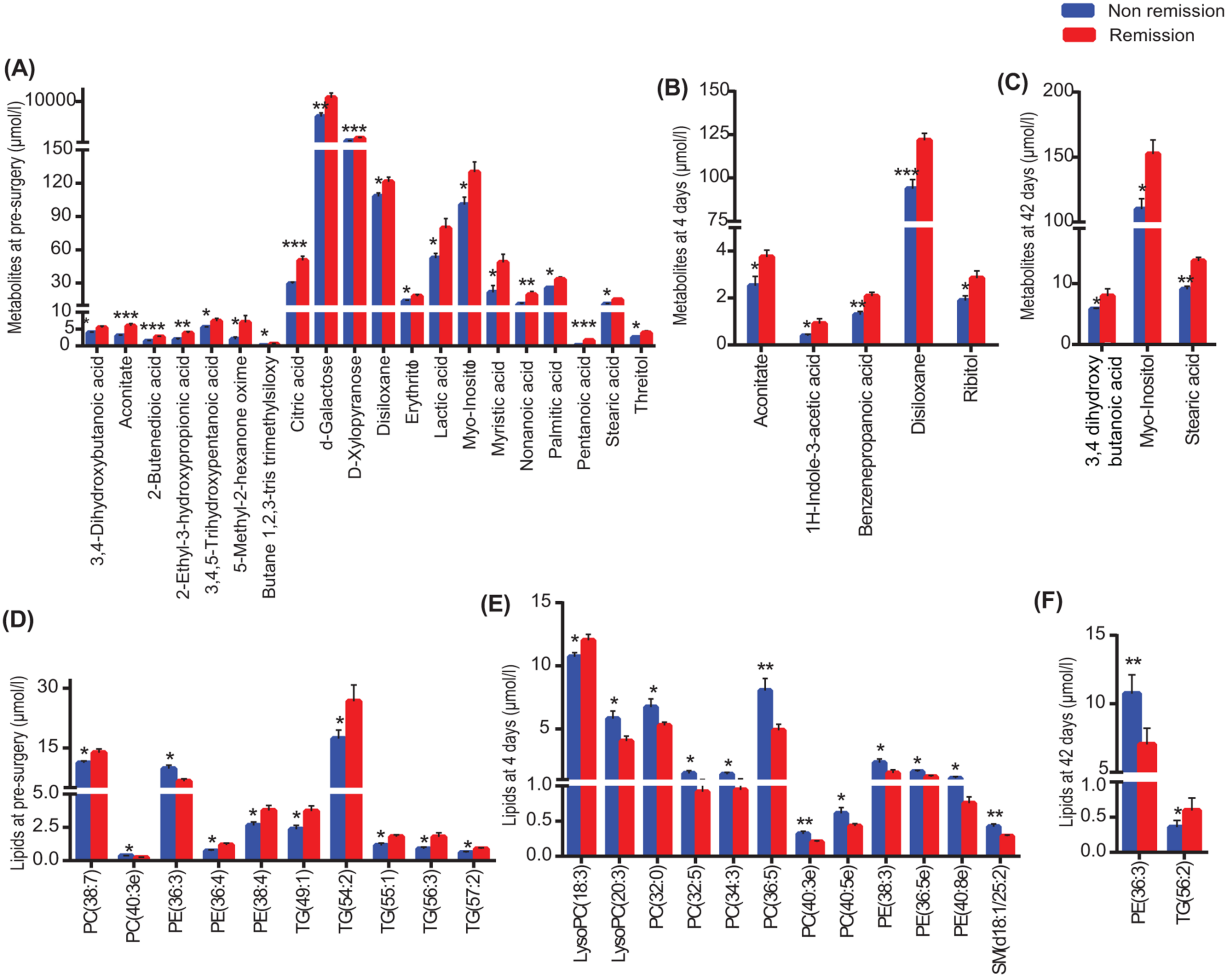
Metabolites at (A) pre (B) 4 days and (C) 42 days after surgery and lipids at (D) pre (E) 4 days and (F) 42 days after surgery that differed significantly between patients in diabetes remission and not in remission. *p<0.05; **p<0.01; ***p<0.001. LysoPC = lysophosphatidylcholine; N-REM = patients not in diabetes remission; PC = phosphatidylcholine; PE = phosphatidylethanolamine; REM = patients in diabetes remission; SM = sphingomyelin; TG = triglycerides.

We compared levels of metabolites and lipid species measured pre-surgery and 4 and 42 days after surgery between subjects in remission and not in remission 2 years after surgery to identify potential variables that may help explain the differences in diabetes remission. Pre-surgery levels of the metabolites belonging to TCA cycle and pentose phosphate pathway were significantly higher in REM versus N-REM subjects (Fig 3A). The differences in metabolites at 4 days after surgery included higher levels of aconitate, indole acetic acid and ribitol that were higher in REM subjects (Fig 3B). At 42 days after surgery, REM subjects had higher levels of 3, 4 dihydroxybutanoic acid, myo-inositol and stearic acid (Fig 3C). Pre-surgery levels of lipid species that were higher in REM versus N-REM patients included TGs with long-chain fatty acids (Fig 3D). At 4 days after surgery, both PC and PE species were lower in REM versus N-REM subjects (Fig 3E). At 42 days after surgery, PE (36:3) levels were lower and TG (56:2) levels were higher in REM versus N-REM subjects (Fig 3F).

## Discussion

Global lipidomics and metabolomics of biofluids provide identification of molecular profiles associated with disease progression and may help to determine biomarkers of disease in the clinical setting [18,19]. In this study, we showed that RYGB promoted early changes in metabolomic and lipidomic profiles and showed that subjects in diabetes remission were characterized by higher pre-surgery levels of metabolites belonging to central carbon pathways and TGs with long-chain fatty acids.

A clear separation in PLS-DA plots was observed between the subjects at pre-surgery and 42 days after surgery for both the metabolomic and lipidomic profiles. At 4 days, there was no clear separation between subjects, which may indicate the effect of general surgery and acute caloric restriction on the metabolic profiles. The top variables determining clustering were the MCFAs octanoic and decanoic acids, which were increased after surgery. MCFAs from TGs are released directly into portal vein after absorption in enterocytes and are transported to liver for rapid metabolism [20]. Incorporation of TGs containing MCFAs were shown to increase total energy absorption in patients with small bowel resection and intact colon [21], situations that are similar to gastric bypass. Aromatic amino acid (tyrosine) and metabolites of aromatic amino acid (benzeneacetic acid) and BCAA (4-methyl oxovaleric acid and 2-ethyl-3-hydroxy propionic acid) correlated positively with insulin levels at 4 days after surgery. Levels of BCAAs, their methylbutyryl and isovaleryl carnitines, and aromatic amino acids have been reported earlier to correlate positively with the diabetic phenotype [22–24] and to decrease after RYGB [7,10,25].

We observed a sharp reduction in most of the top lipid species at 4 days followed by an increase at 42 days after surgery, which might reflect lipid remodeling resulting from the surgical process. A sharp reduction in total TGs and cholesterol within 1 day of biliopancreatic diversion has previously been reported [26]. TGs with long-chain fatty acids correlated negatively with insulin levels at 4 days after surgery, whereas short-chain TGs correlated positively with insulin levels 42 days after surgery. Previous studies have shown that higher chain length and unsaturation in TGs and phospholipids correlate with reduced diabetes risk in humans [27] and increase after weight loss [28]. Furthermore, a reduction in short-chain TGs has been reported previously 3–6 months after RYGB [29] and we observed decreased levels of short-chain TGs at early time points after surgery. Together, these findings suggest that altered levels of short-chain TGs may contribute to metabolic improvement after surgery.

We also compared levels of metabolites and lipids between REM and N-REM subjects and observed that subjects in diabetes remission 2 years after surgery had elevated pre-surgery levels of metabolites related to central carbon metabolism such as citric acid, fumarate and aconitate (from the TCA cycle) and xylopyranose, erythritol and threitol (sugars and polyols from the pentose phosphate pathway). Impaired flux of TCA cycle intermediates in diabetes is associated with insulin resistance [30]. In addition, pre-surgery levels of TGs with long-chain fatty acids (54:2, 55:1, 56:3 and 57:2) and phospholipids with long-chain fatty acids [PC (38:7) and PE (36:4, 38:4)] were higher in subjects with diabetes remission, consistent with previous reports correlating elevated levels of lipid species with higher chain length with reduced diabetes risk [27,28]. We observed higher levels of aconitate and ribitol at 4 days after surgery in REM versus N-REM subjects. These factors belong to central metabolic pathways involved in energy generation, and may thus potentially contribute to the improvement in diabetes remission. There were no clear differences in metabolites and lipid species between REM and N-REM subjects at 42 days that could explain later differences in diabetes status.

It is important to emphasize that the low number of patients in the current study limits the number of correlations that can be performed. Lack of a non-diabetic control group who did not undergo surgery prevents comparison of lipid and metabolite status between operated and non-operated subjects. A previous study suggests the importance of caloric restriction in the improvement of glucose homeostasis at early time points after RYGB [5]. However, lack of information on the food intake in our study limits the interpretation of results at 4 days after surgery. Furthermore, we cannot exclude the possibility that changes observed 4 days after surgery may be the result of the procedure rather than metabolic adaptations. Thus, these observations require further investigation in a larger cohort and mechanistic studies to elucidate if the observed differences in metabolites and lipids contribute to diabetic remission after RYGB.

## Conclusions

We report early alterations in the metabolome and lipidome after gastric bypass in insulin-resistant morbidly obese subjects. The beneficial effects of surgery included a reduction in BCAA metabolites and short-chain TGs, which were observed as early as 4 days after surgery. It also seems that subjects with diabetes remission were more metabolically active than subjects who were not in diabetes remission.
